# Mesenchymal Migration as a Therapeutic Target in Glioblastoma

**DOI:** 10.1155/2010/430142

**Published:** 2010-06-21

**Authors:** Jessie Zhong, Andre Paul, Stewart J. Kellie, Geraldine M. O'Neill

**Affiliations:** ^1^Children's Cancer Research Unit, Kids Research Institute, The Children's Hospital at Westmead, Locked Bag 4001, Westmead, NSW 2145, Australia; ^2^Discipline of Paediatrics and Child Health, The University of Sydney, Sydney, NSW 2006, Australia; ^3^Oncology Department, The Children's Hospital at Westmead, Westmead, NSW 2145, Australia

## Abstract

Extensive infiltration of the surrounding healthy brain tissue is a cardinal feature of glioblastomas, highly lethal brain tumors. Deep infiltration by the glioblastoma cells renders complete surgical excision difficult and contemporary adjuvant therapies have had little impact on long-term survival. Thus, deep infiltration and resistance to irradiation and chemotherapy remain a major cause of patient mortality. Modern therapies specifically targeted to this unique aspect of glioblastoma cell biology hold significant promise to substantially improve survival rates for glioblastoma patients. In the present paper, we focus on the role of adhesion signaling molecules and the actin cytoskeleton in the mesenchymal mode of motility that characterizes invading glioblastoma cells. We then review current approaches to targeting these elements of the glioblastoma cell migration machinery and discuss other aspects of cell migration that may improve the treatment of infiltrating glioblastoma.

## 1. Introduction

Glioblastoma brain tumors are the most common adult brain cancer and have a very poor prognosis. Despite intensive efforts, there has been little improvement in the ability to successfully treat these tumors, and a major reason for this failure is the unique ability of the glioblastoma cells to extensively spread throughout the surrounding healthy brain tissue. There is increasing realization that the molecular regulators of glioblastoma invasion may be key to the development of new therapeutic approaches [1]. Developments in the field of cell migration have led to the appreciation that invading cancer cells can adopt different modes of cell migration, and the glioblastoma cells appear to specifically use a mesenchymal mode of cell migration. In the present paper, we focus on the adhesion signaling networks and actin cytoskeleton dynamics that are implicated in mesenchymal migration and discuss how these molecules represent exciting potential targets for therapeutic arrest of glioblastoma cell invasion.

### 1.1. Glioblastoma Survival and Prognosis

Gliomas are a class of primary tumors that arise in the brain, and the most common and lethal form is the grade IV glioblastomas (previously known as glioblastoma multiformae) [2]. While brain cancers generally have lower incidence than other more prevalent cancers—similar to rates worldwide, brain cancers accounted for ~2.5% of cancer deaths in the state of New South Wales, Australia in 2006—there has been little improvement in patient survival despite advances in technology, surgery, and adjuvant therapies over the last two decades. Patient prognosis is dismal with almost 100% rate of final mortality [3]. The median survival time for patients with glioblastoma remains only 12–15 months [4, 5], and the 3% 5-year survival rate is significantly worse than the 60% survival rate seen for other brain tumors such as oligodendroglioma and medulloblastoma [5, 6]. Moreover, survival rates for glioblastoma stand in stark contrast to the high survival rates for other more common cancers such as prostate (88%), breast (88%), colon (63%), and melanoma (80%) [5].

The low survival rates for glioblastoma are, in part, a consequence of the extensive infiltration of healthy brain tissue that is a cardinal feature of these tumors. Diffuse infiltration throughout the brain makes these tumors refractory to successful surgical excision, and tumor recurrence is almost inevitable, with 90% of patients developing new lesions within 2-3 cm of the original site [7] or at distant sites in the brain [8]. Notably, despite extensive infiltration of the surrounding healthy brain tissue, the glioblastomas rarely metastasize outside the brain and the infiltration of the brain tissue is significantly determined by interaction between the glioblastoma cells and the unique extracellular brain environment. A number of extracellular matrix (ECM) proteins (such as hyaluron, vitronectin, tenascin-C, osteopontin, and SPARC) are upregulated at the edge of the advancing glioblastoma tumor, and this may alter cell invasion (reviewed in [9]). In addition, glioma cell adhesion is enhanced in regions of the brain where ECM proteins are present, such as in the blood vessels, and it suggested that this may facilitate glioblastoma invasion [10, 11].

### 1.2. Glioblastoma Characteristics and Diagnosis

Glioblastomas are characterized histopathologically by diffuse infiltration, increased cellular proliferation, increased angiogenesis, nuclear atypia, and necrosis [2], and tumors with these characteristics are categorized as a grade IV glioma by the World Health Organization (WHO) [12]. Glioblastomas may be tumors of either *de novo* origin or may develop from a low grade glioma but are histopathologically indistinguishable [13]. However, they have distinct genetic profiles, and there are distinct treatment implications for these two different tumor categories [14].

Primary or *de novo* glioblastoma accounts for approximately 90% of glioblastoma and is chiefly diagnosed in older patients with a mean age of 62 years [13]. Genetic changes characteristically associated with primary glioblastoma include amplification and/or overexpression of Epidermal Growth Factor Receptor (EGFR) (~60%) [15, 16] and Mouse double minute 2 (Mdm2) (a key negative regulator of the tumor suppressor p53) [17, 18], deletion mutations of Cyclin-Dependent Kinase inhibitor 2A (CDKN2A, also known as p16INK4A, a cell cycle regulator) [19], and inactivating mutations of Phosphatase and Tensin Homolog Deleted on Chromosome 10 (PTEN) [20, 21]. EGFR and PTEN gene mutations are likely to play a role in glioblastoma invasion based on the known interaction between EGFR and Focal Adhesion Kinase (FAK) to promote cell migration [22] and the role for PTEN as an inhibitor of cell migration [23, 24]. The potential role, if any, of the other characteristic mutations in invasion is presently unknown.

Another group of glioblastomas is known to arise from previously identified gliomas, usually developing as a result of malignant transformation several years after initial diagnosis of a low grade astrocytoma [25]. This type of glioblastoma is relatively rare by contrast and occurs in younger patients with a mean age of 45 years [13]. Given that mutations of the tumor suppressor p53 are present in two-thirds of low grade astrocytomas, it is not surprising that p53 is the predominant genetic aberration in secondary glioblastomas (~60%) [26–28]. Other less frequent defects include amplification/overexpression of Platelet Derived Growth Factor Receptor (PDGFR, a receptor tyrosine kinase involved in proliferation, migration, and angiogenesis), deletion of Retinoblastoma gene (RB), and loss of heterozygosity at 17p, 19q, and 10q [29, 30]. O^6^-methylguanine-DNA methyltransferase (MGMT) is a DNA repair enzyme that protects cells by removing alkyl groups from the O^6^ position of guanine. The activity of MGMT reduces the cytotoxicity of alkylating chemotherapy agents (such as temozolomide) and contributes to drug resistance by reversing the DNA damage induced by such agents [31], and therefore, MGMT silencing is a favorable marker. Epigenetic silencing of MGMT by promoter methylation has been detected in 36% of primary glioblastomas, 75% of secondary glioblastomas, and 40% of pediatric cases [31, 32] and confers a better prognosis for both adult and pediatric patients who receive temozolomide treatment [32–34].

The brain tumors are one of the few classes of solid tumor observed in both pediatric and adult patient populations. Interestingly, the trend for incidence of the different grades of brain tumor tends to be reversed in the pediatric population with the grade IV glioblastomas being less common (CBTRUS, 2002–2006). However, the 5-year survival rate for pediatric patients is only 5–15% [35], and thus the outlook for children diagnosed with glioblastoma remains poor. Pediatric glioblastomas have a pattern of genetic modifications distinct from that in adults. Although overexpression of the EGFR protein is observed in 40% of grade IV pediatric gliomas, EGFR gene amplification in children is very rare [36]. p53 gene mutations are very frequent, occurring in 33% of pediatric glioblastomas, and 50% of these patients overexpress mutant p53 protein [35]. p53 overexpression increases with tumor grade and is correlated with patient outcome [37]. Unlike the adult tumors, very little information is available about RB expression in childhood glioblastoma astrocytoma [25]. Knowledge of the molecular regulation of pediatric glioblastoma is significantly less than that for the adult form of the disease, and there is an urgent need to develop cell culture models derived from pediatric tumor material to address this imbalance.

## 2. Mechanisms of Glioblastoma Infiltration

Most solid cancers progress to disseminated metastatic disease, evidenced by secondary tumors arising in sites distal to the primary tumor. The rarity of glioblastoma spread outside the brain reflects the important interaction between the glioblastoma cells and the unique brain environment. Thus, the mechanisms of glioblastoma infiltration represent a potentially specific target for treating glioblastoma.

### 2.1. Glioblastomas Use a Mesenchymal Mode of Migration and Invasion

**Figure 1:**
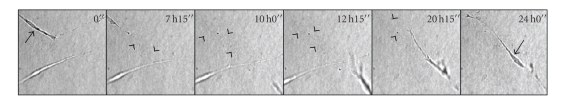
Glioblastoma cells follow the tracks created by other cells in 3D collagen gels. U87-MG glioblastoma cells were seeded into collagen type I gels for 24 hours, and then 24-hour time-lapse imaging was performed to track cell migration. Arrows (↑) indicate a “leading” cell and a “follower” cell, and arrows heads (∧) indicate the single path that they both follow in the collagen gel.

**Figure 2:**
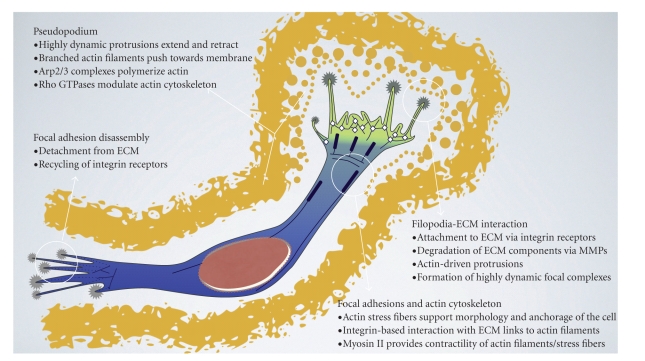
Schematic representation of a glioblastoma cell migrating through a 3D ECM. During migration, the cell becomes polarized with respect to the direction of movement into a leading and a trailing edge. The leading edge is characterized by dynamic membrane rearrangements and proteolytic breakdown of ECM, enabling the cell to protrude at its front. The trailing edge displays constant disassembly of mature focal adhesions, therefore promoting dislodgement of the rear. Tight regulation of actin assembly and disassembly is crucial for migration, controlling cellular protrusion, as well as myosin II-mediated contraction in interplay with myosin II.

The use of 3-dimensional (3D) collagen gels, organotypic brain slice cultures, and imaging of fluorescently labeled glioblastoma cells in *in vivo *mouse models has established that glioblastoma cells migrate individually with a mesenchymal mode of motility [38–40]. This is typified by a polarized extension of leading edge membrane processes in the direction of cell migration. This process is critically dependent on the regulated formation and disassembly of transmembrane integrin receptor-mediated adhesions to the extracellular matrix, known as focal adhesions [41]. Characteristically during mesenchymal migration, cells secrete matrix metalloproteases (MMPs) at the leading edge that break up extracellular components to create corridors for migration [41, 42]. In the intact brain the glioblastoma cells travel along white matter tracks and basement membranes lining blood vessels [43], and thus, the brain environment uniquely advantages migration of the glioblastoma cells. In 3D collagen gels we have observed a “leader” glioblastoma cell that appears to reorganize the collagen and create a track which subsequent glioblastoma cells then follow (Figure 1); the ability of glioblastoma cells to reorganize the extracellular matrix is critical to the ability of the cells to disseminate throughout the brain [1]. Understanding how the glioblastoma cells interact with and reorganize the extracellular environment will provide much needed new approaches to specifically targeting glioblastoma. During mesenchymal migration through a 3D matrix, cells protrude a leading pseudopodium with short-lived actin-rich membrane protrusions as well as long-lived finger-like protrusions of up to 50 *μ*m length, termed filopodia (Figure 2). These structures are followed by focal adhesion formation at the cell front and subsequent detachment of adhesive contacts at the rear of the cell [40, 44, 45]. This is a highly dynamic process with paxillin-containing adhesions at the base of protrusions that disassemble as new adhesions form near the leading edge—a process referred to as adhesion turnover [46]. A hallmark of glioblastoma cell infiltration through a 3D environment is the extension of long, thin polarized membrane extensions that explore and penetrate the surrounding environment [38, 39]. Presumably cells employ this mechanism *in vivo *to find and then travel along the white matter tracts and endothelial lining of blood vessels in the brain.

### 2.2. Integrin Receptors and Focal Adhesions in Mesenchymal Migration

There are intensive research efforts currently underway to try and understand the critical molecular regulators of mesenchymal migration. Although our understanding of this process remains incomplete, it is useful to understand the present state of play and consider how this currently and, may in the future, informs therapeutic approaches to the treatment of glioblastoma. The focal adhesions (FAs) are points of linkage between the ECM, the transmembrane integrin receptors, and the internal actin cytoskeleton. In addition to their role as anchorage points to the matrix, the FAs also transmit information bidirectionally between the cell and the external environment [47], and the regulated formation and disassembly of these sites, is critical to mesenchymal migration. 

The integrin receptors are heterodimeric transmembrane complexes consisting of an *α* and a *β* subunit [48]. Mammals utilize 18 *α* and 8 *β* subunits, and combinations of these subunits create receptors for specific ECM components including fibronectins, laminins, and collagens [49–51]. During tumor development, changes in integrin receptor expression, intracellular control of integrin function, and signals perceived from integrin receptor ligand binding influence the cell's ability to interact with the environment, enabling metastatic cells to convert from a sessile, stationary phenotype to a migratory and invasive phenotype [51, 52]. Although there is some evidence for elevated expression levels of distinct integrin receptor subunits (*α*v, *β*1, and *β*3) in cell lines derived from disseminated cancers [53], there is no subunit expression profile that can function as a marker of metastasis [54]. However, integrin receptor *α*v*β*3 is highly expressed on the cell surface across a range of tumor types, including glioblastoma [55]. The *α*v*β*3 receptor is an important attachment factor for ECM proteins with the exposed arginine-glycine-aspartate (RGD) motif such as fibronectin [56–58]. Much focus has been placed on the role of the *α*v*β*3 receptor in the angiogenesis that is characteristic of the highly vascularized glioblastomas [59]. However, equally important but less emphasized to date is the role of *α*v*β*3 in mesenchymal cell migration. This receptor is found in the initial rapidly turned over adhesions (focal complexes) that form at the migrating cells leading edge [60] and may be important in determining the formation of other adhesion types in the cell [61]. Given that the *α*v*β*3 receptor is highly expressed in glioblastoma [62–67] and indeed is a target of major new therapies (see details below), it is paramount that trials designed to target this receptor in glioblastoma consider the role this receptor plays not only in glioblastoma neo-angiogenesis but also in the invasion of glioblastoma cells.

### 2.3. The FAK/Src Signaling Axis in Mesenchymal Migration

**Figure 3:**
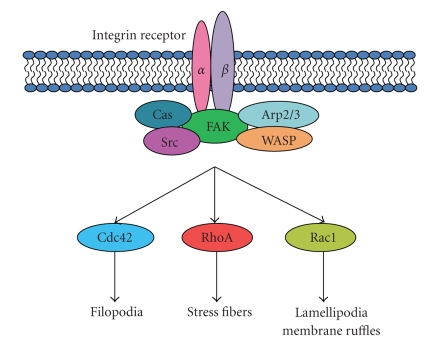
The FAK/Src/Cas signaling network downstream from integrin receptor engagement. Downstream from this network, RhoA, Rac1, and Cdc42 induce cytoskeletal changes that regulate cell migration.

Components of the Focal Adhesion Kinase (FAK)/Src tyrosine kinase migration signaling network (Figure 3) are upregulated and activated in glioblastoma and a number of therapeutic approaches targeting these molecules are currently under clinical trial. Gliomas express elevated expression levels of the nonreceptor tyrosine kinase FAK, and this is particularly true of cells at the invasive margins of the primary tumor [68]. Moreover, FAK signaling appears critical to the migration of glioblastoma cells [69, 70]. The proto-oncogene Src tyrosine kinase is a major FAK interactor; interaction of these two proteins is a vital determinant of mesenchymal cell migration. Both FAK and Src have emerged as important targets for the treatment of glioblastoma [71]; thus below we describe the FAK/Src signaling axis in mesenchymal migration. 

Integrin receptors lack any intrinsic catalytic activity and instead function by recruiting an array of cytoplasmic proteins [72] which in turn establishes phosphorylation-dependent signaling networks. FAK functions as an integrin-activated “scaffold” for the recruitment of signaling proteins that contain Src homology (SH) domains, SH2, and SH3, to sites of integrin receptor clustering [73], and is involved in the dynamic regulation of actin and focal adhesion structures [74]. Genetic ablation of FAK reduces mesenchymal cell migration [75], and conversely, elevated FAK expression enhances cell migration in an Src/Fyn-dependent manner [76, 77]. Not surprisingly therefore, high FAK expression levels are observed in a range of invasive human tumors [78]. Current models of FAK activation suggest that intramolecular interactions between FAK domains inhibit the protein's enzymatic function, and this inhibition is reversed following receptor stimulation, leading to FAK autophosphorylation at tyrosine 397 [79]. This exposes binding sites for SH2 and SH3 domains of Src kinase. Bound Src then catalyzes the phosphorylation of tyrosines 576 and 577 in the activation loop of FAK, conferring full catalytic activity to the enzyme. FAK activation and/or phosphorylation is critical for promoting cell migration [23, 24, 73, 80, 81]. Activated FAK binds and phosphorylates a number of signaling proteins by recognizing SH2 and SH3 domains including Shc [82], the p85 subunit of phosphatidylinositol 3-kinase (PI3K) [83], the Rho GTPase activating protein GRAF [84], growth factor receptor bound protein 2 (Grb2), paxillin [85], and members of the Cas family of adhesion docking proteins [77, 86–88]. 

Importantly, a second FAK subfamily member, Related Adhesion Focal Tyrosine Kinase (RAFTK)/Proline-rich tyrosine kinase (Pyk2), may also play a role in glioblastoma infiltration. These two proteins have overlapping functions and downstream partners, and while FAK is ubiquitously expressed, RAFTK/Pyk2 has a more restricted expression and is predominantly seen in brain and hematopoietic cells. RAFTK/Pyk2 is correlated with increased malignancy in glioblastoma [89], and overexpression accelerates cell invasion in breast cancer [90]. Critically, both FAK and RAFTK/Pyk2 knockdowns enhanced survival in a mouse model of orthotopic glioma xenografts [91]. Thus both FAK and RAFTK/Pyk represent important targets for arresting glioblastoma infiltration.

Src is similarly activated in numerous tumor types [92], and the kinase activity of this protein is required for focal adhesion turnover during migration [93]. Among proteins of the adhesome, Src displays the greatest number of interactions with other molecules in that network [72] and thus, presumably, is a key regulator of the focal adhesion signaling networks. Multiprotein complexes consisting of interactions between active FAK, Src, and adaptor molecules such as paxillin and the Cas family of proteins are important determinants of downstream signaling to promote cell migration. Within these complexes, phosphorylation of paxillin and the Cas proteins by Src induces cell migration [94–97]. Indeed, it is suggested that FAK serves as a scaffold for Src and the Cas protein p130Cas and that this molecular interaction results in sustained Src signaling [87, 98, 99]. 

Via this activity of FAK and Src and their target molecules, including those of the Cas family of proteins and paxillin, a signaling network is established that culminates in the activation of GTPase proteins, such as Rac. In turn, this determines the dynamic state of the actin cytoskeleton that is essential to the morphological progression of mesenchymal cell migration [77, 100, 101]. The small GTPase Rac is a specific regulator of mesenchymal cell migration [102] that stimulates the branching of short actin filaments at the cell's leading edge. The newly polymerized short filaments push on the membrane to form the leading edge protrusion that is a characteristic feature of mesenchymal cells. By employment of such phosphorylation cascades, the cell is responsive to extracellular cues in a tightly regulated manner by reorganization of the actin cytoskeleton, resulting in either cell migration or adhesion [77, 103–105].

### 2.4. Actin Function and Regulation During Mesenchymal Migration

Cells migrating with a mesenchymal phenotype have a characteristic array of polymerized actin, displaying short, branched filaments at the leading edge and longer tension-bearing filaments in the cytoplasm known as stress fibers [40]. Directed movement is facilitated by complementary effects of the FA (sensing and attaching to ECM) and the actin cytoskeleton (mediating cell shape). Actin filaments are physically linked to the FA through molecules, such as talin and vinculin that contain both integrin and actin binding domains. When connected to the FA, the stress fibers become contractile due to the integration of myosin II [106–109], and the contraction of such actomyosin filaments is the basis for cell locomotion. Myosin II contraction is achieved by phosphorylation of the myosin light chain via the Ca^+2^- and calmodulin-dependent myosin light chain kinase (MLCK) [110]. Desphosphorylation of the myosin light chain by the MLC phosphatase (MLCP) results in myosin II inactivation. The effects of MLCP can be countered by phosphorylation mediated by the Rho GTPase effector Rho-kinase [108, 111].

Given the critical role of FA and actin interaction in cell migration, much research has concentrated on the mechanistic relationship between actin nucleators and FA proteins in the formation of new actin structures [112]. The actin nucleation process is mediated by the actin-regulated protein 2/3 complex (Arp2/3) and activated by the Wiskott-Aldrich syndrome protein (WASP) and WASP family Verprolin-homologous (WAVE) family of proteins [112–114]. As a result of WASP activation, actin polymerization pushes the plasma membrane forward, leading to ruffling and pseudopodial extension of the leading edge cell membrane [115, 116]. In 2006, Butler et al. reported that purified *α*v*β*3 integrin receptor complexes exhibit enhanced actin polymerization activity, thus providing evidence to suggest a direct interaction between actin nucleation and adhesion sites [117]. Although the focal adhesion protein vinculin transiently associates with Arp2/3 during cell adhesion to fibronectin or after epidermal growth factor stimulation [118], the fact that vinculin-negative cells can still generate lamellipodia and migrate faster [119, 120] suggests that vinculin may not be the major adaptor candidate for integrin-actin signaling. The Arp2/3-WASP complex directly interacts with FAK, but only at sites where the latter is not active (not phosphorylated at tyrosine 397), for example, in mature adhesion structures [121]. These data give rise to the conclusion that FAK interaction with Arp2/3 may regulate the formation of early protrusive lamellipodia [121], and therefore FAK represents an excellent target for blocking the migration of glioblastoma cells.

### 2.5. Rho GTPases and Mesenchymal Cell Migration

The driving force for cell movement is derived primarily from the coordinated assembly and disassembly of actin filaments and the Rho family GTPases, RhoA, Rac1, and Cdc42 are critical regulators of this process. Together these enzymes regulate the organization of actin filaments and cooperate to control polarity, protrusion and adhesion during cell movement [113, 122]. Other Rho GTPases, such as RhoG, RhoD, TC10, and Rif (RhoF) can also induce actin-based protrusions at the cell membrane [123–125]. Activity of RhoA, Rac1, and Cdc42 is associated with distinct populations of actin filaments and associated adhesions [126]. Rac1 activation promotes the formation of precursor adhesions (focal complexes) in the meshwork of actin filaments at the leading edge [60] while Cdc42 stimulates small focal complexes at the tip of thin membrane protrusions, known as filopodia, that contain parallel bundles of actin filaments [115]. RhoA activation is associated with the formation of mature focal adhesions and actin stress fibers [127, 128]. RhoA, Rac1, and Cdc42 activate WASP proteins and Diaphanous-related formins (DRFs) that in turn promote actin polymerization. Importantly, Cdc42 and Rac1 are active at the leading edge of a migrating cell, where their targets, WAVE/N-WASP, are located [114]. Rac1 is a key regulator of migration and localizes to the leading edge of a moving cell where it is activated by growth factors and integrin-mediated cell adhesion [129, 130]. Rac1 activation is a major target of signaling through the Cas family of proteins [101, 131, 132]. A number of studies have indicated that Rac1 is a specific regulator of mesenchymal migration, and indeed Rac1-dependent invasion through a 3D matrix is one of the defining features of mesenchymal cell migration [41, 102, 133, 134]. 

Rho proteins can also regulate the actin depolymerizing factor ADF/cofilin, and thus actin nucleation, indirectly through Rho kinase (ROCK) and p21-activated kinase-1 (PAK-1). RhoA activates ROCK while Cdc42/Rac1 activates PAK-1/-2/-3. ROCK and PAK in turn phosphorylate and activate LIM-motif-containing kinase protein (LIMK), which inactivates cofilin and thus the recycling of actin filaments at early lamellipodial extensions [113, 135–137].

## 3. Current Therapeutic Approaches to Targeting Infiltrating Glioblastoma

The history of treating malignant gliomas dates back over a century. The first surgical operation to treat brain cancer was reported in 1884 but barely made an improvement on patient survival [138]. In the early to mid 1900s, surgeons performed hemispherectomies (surgical removal of a cerebral hemisphere) in a desperate bid to cure patients with glioblastoma, despite the inevitable consequences of hemiplegia (paralysis of one side of the body) and hemiparesis (weakness on one side of the body) [139]. In spite of the high cost of such a radical treatment, hemispherectomy could not guarantee full removal of the glioblastoma cells. The subsequent introduction of radiation therapy prolonged survival by several months but was still unsuccessful as a long-term treatment [140]. Finally, chemotherapy was developed, but although combined chemotherapy and radiation therapy has significantly improved patient survival for various solid tumors, it is still incapable of curing most glioblastoma patients in the long term [4]. 

The standard treatment for newly diagnosed glioblastomas consists of initial surgery to remove as much of the tumor as possible, followed by radiation therapy and chemotherapy [141]. Surgical resection, the first step, eradicates as much of the tumor as possible, and studies have suggested a positive correlation between aggressive surgical resection and survival [142]. However, in 35–40% of patients, an attempt at surgical resection is not an option due to the medical condition of the patient or more often because of the location of the tumor [143]. In these cases, patients may resort to stereotactic radiosurgery, a form of intense and localized radiation therapy. Previously, standard chemotherapy for both adult and pediatric glioblastoma patients involved the administration of PVC (procarbazine, vincristine, and lomustine (CCNU)) or, alternatively, nitrosourea drugs such as carmustine (BCNU) and CCNU. More recently, the use of the oral alkylating drug temozolomide is growing and has become the established adjuvant standard of care because it has good ability to cross the blood brain barrier [144] and has proven to be more beneficial than the traditional chemotherapy agents [145, 146]. However, despite prolonging the time to progression, temozolomide does not significantly extend overall survival [147]. This relative lack of success in glioblastoma treatment throughout history has highlighted the need to develop new, more effective therapies. One popular target for therapy is angiogenesis, the process of blood vessel formation, which is a critical factor in glioblastoma invasion as well as in other types of cancers [148]. However, glioblastomas can also incorporate pre-existing vasculature (known as cooption) as an alternative to forming new blood vessels (neovascularization) [149]. *In vivo* imaging of glioma cell invasion has revealed that the main tumor mass can grow by cooption [150] and that cooption can occur in response to angiogenesis inhibition [151]. These findings were confirmed in clinical trials of antiangiogenic agents, whereby a subset of glioblastoma patients treated with bevacizumab (a Vascular Endothelial Growth Factor-targeting agent) experienced tumor recurrence with a more infiltrative phenotype that resembled gliomatosis (a type of malignant glioma with extreme infiltrative capacity) [152]. This suggests that blocking angiogenesis may force the cells into the alternative cooption pathway. Thus, it is critical to consider other glioblastoma signaling pathways that may be targeted; below we consider those treatments targeting molecules that are prominent players in migration.

### 3.1. Agents That Target Integrin Receptors

Integrin receptors reported to be upregulated on glioma cells include *α*3*β*1, *α*v*β*1, *α*v*β*3, and *α*v*β*5 [153] although *α*v*β*3 expression levels can differ across glioma cell lines [154]. The *α*v*β*3 and *α*v*β*5 receptors are expressed both in endothelial cells associated with new vasculature and in the glioma tumor cells [59, 155–157] and increased expression of *α*v*β*3 has been observed in angiogenic endothelial cells in tumors [158] and is linked to more aggressive, metastatic breast cancers [159, 160]. The ability for tumors to grow past a certain size depends on the ability of the tumor to establish its own blood supply via the process of angiogenesis [161], and there has therefore been a major worldwide effort to target the molecular regulators of angiogenesis. The *α*v*β*3 receptor plays a vital role in angiogenesis [59, 162], and the agent cilengitide (EMD121974) was synthesized to target the RGD motif that is recognized by integrin receptors and thus inhibits *α*v*β*3-dependent adhesion [163]. Notably, this compound also targets the *α*v*β*5 receptor as both of these integrins interact with their ligands via specific RGD motifs [164]. Importantly, despite originally being designed as an antiangiogenic therapy, cilengitide has a range of effects *in vitro*, including apoptosis and blocking cell adhesion, migration, and invasion [165]. In preclinical studies using an orthotopic xenograft mouse model with the human glioblastoma cell line U87MG, cilengitide improved survival time and inhibited tumor growth and angiogenesis [166]. Furthermore, cilengitide can inhibit glioma cell proliferation at concentrations as low as 1 *μ*g/mL while 50 *μ*g/mL can induce significant apoptosis [165]. Cells treated with cilengitide display disassembly of actin filaments and loss of cell-cell contacts [165], dosage-dependent inhibition of FAK and Src activity [165], and detachment-induced apoptosis [167, 168]. Phase I and II clinical trials in glioma patients have had promising results, especially for patients with hypermethylated MGMT [169, 170], and cilengitide is currently in Phase III trials in glioblastoma patients treated with radiation therapy and chemotherapy [171]. The drug is well tolerated, with patients suffering only minimal side effects [170, 172], and the efficacy is reported to increase when used in combination with radioimmunotherapy or temozolomide [62, 173].

### 3.2. Targeting Cytoplasmic Adhesion Molecules

TAE226 is a novel small molecule designed to inhibit FAK by blocking phosphorylation of FAK tyrosine 397. The mechanism of action is specific for FAK autophosphorylation as it does not affect total FAK protein expression and has no effect on either EGF-induced EGFR phosphorylation or serum-induced PDGFR phosphorylation [174]. TAE226 has yet to be tested in clinical trials for glioblastoma, but *in vitro* experiments demonstrated increased apoptosis and decreased angiogenesis [175]. In studies of neuroblastoma, TAE226 induced apoptosis and G2 cell cycle arrest and compromises cell viability [176] while in trials in ovarian cancer, the drug exerts a number of anticancer effects, including reduction in tumor burden, angiogenesis, and cell proliferation and improves patient survival [177].

Inhibition of Src activity in either two-dimensional (2D) or 3D culture models can successfully block glioblastoma invasion [178, 179]. For example, Src inhibition impairs pseudopodium formation and actin bursting at the tip of the pseudopodium [178]. Dasatinib is an orally available drug that inhibits Src kinase (as well as Eph receptors, Bcr-Abl, PDGFR*β*, and Kit) [180]. This agent has approval by the Food and Drug Adminstration USA (FDA) for use in treating leukemias and is presently being tested in a number of clinical trials for the treatment of glioblastoma. A Phase I trial is currently investigating the combination with erlotinib (an EGFR inhibitor), while Phase I/II trials to assess the use of a dasatinib/radiotherapy/temozolomide regime in conjunction with adjuvant dasatinib and temozolomide for newly diagnosed glioblastoma are about to be launched [71]. Preclinical studies of dasatinib have already shown successful inhibition of Src activity [180], and an additive effect is observed when combined with radiotherapy or temozolomide [181]. Furthermore, low concentrations of dasatinib can block proliferation of glioma cells *in vitro* [180].

Similar to Dasatinib, AZD0530 is also an orally available drug that inhibits Src. Although still in the preclinical stage of testing for glioblastoma treatment, AZD0530 has been found to inhibit both FAK and paxillin phosphorylation and activity. The resulting abrogation of adhesion-dependent signaling pathways consequently inhibits cell migration [182]. In other investigations, AZD0530 blocks invasion and increases the sensitivity of lung cancer cells to radiation therapy [183], and patient recruitment is in process for Phase II testing in hormone receptor-negative metastatic breast cancer.

### 3.3. Regulators of Actin Cytoskeletal Dynamics

The mammalian target of rapamycin (mTOR) is a protein kinase upstream of PI3K/Akt signaling pathway, and activity of this protein is inhibited by rapamycin. Involved in various signaling pathways implicated in glioblastoma, mTOR plays a role in a number of cellular processes, including cell proliferation and growth, angiogenesis [184]. Critically, rapamycin blocks F-actin organisation and inhibits phosphorylation of FAK, paxillin, and p130Cas [185]. In preclinical studies, PTEN-deficient tumors showed enhanced sensitivity to mTOR inhibitors, and thus the frequency of inactivating PTEN mutations in glioblastoma suggests that mTOR inhibitors may be used to successfully treat glioblastoma [20, 186]. mTOR inhibitors have tolerable levels of toxicity, are effective in reducing the rate of cell proliferation [187], and improve patient survival rates [188]. Adverse effects reported following rapamycin treatment include hypercholesterolemia and hyperglycemia and, more seriously, the activation of Akt [187, 188].

## 4. Exploiting the Unique Biology of Infiltrating Glioblastoma to Identify New Treatment Targets

The approaches outlined above highlight the potential—and in some cases, success—of targeting adhesion signaling pathways in the treatment of glioblastoma. The highly unique behavior exhibited by the glioblastoma cells in response to the specialized morphology and molecular structure of the brain suggests that there is considerably more scope to target these pathways and derive specific and efficacious new therapies. Below, we consider some of the adhesion signaling pathways and mechanisms that might be amenable to therapeutic exploitation along with some of the envisaged caveats.

### 4.1. Further Targeting of Integrin Receptors

Downregulation of *β*-integrin by antisense *β*-1 mRNA in a rat model of glioma caused a significant reduction of brain invasion, attributed either to impaired interaction with the ECM in the brain or interference with *β*-1-dependent signaling pathways [189]. Similarly, direct inhibition of integrin by an integrin-specific antibody led to a reduced capacity of ovarian tumor cells to proliferate [190]. Studies of *α*v*β*3 integrins in the brain have revealed that the activation state of integrins rather than expression level controls metastasis and angiogenesis in tumor cells, and this process is highly susceptible to cues from the brain microenvironment [191]. Thus, the full potential for therapies targeting integrin receptors in the treatment of glioblastoma has probably not yet been fully realized. However, data suggesting that integrin expression may also be essential for maintaining cells in an attached and stationary mode [192] highlight the need for a thorough understanding of integrin-related pathways.

In recent times, it has become clear that invading and migrating cancer cells have a range of motility modes at their disposal that allows the cells to migrate through distinct extracellular environments [40, 193–195]. The ability of certain cancer cells to switch between an amoeboid-type migration that does not require the action of MMPs versus a mesenchymal-type MMP-dependent migration mode is, at least part of, the reason behind the less than hoped for success using anti-MMP therapies to treat metastatic cancers [40, 196]. In addition to the differential requirement for MMPs, each migration mode is characterized by distinct requirements for integrin-mediated interaction with the ECM; integrin attachment is an essential requirement for mesenchymal cell but of less significance for amoeboid motility. The potential for cancer cells to transition between migration modes has become a confounding factor when targeting anticancer pharmaceuticals to integrins [67, 197]. As reviewed above, studies to date suggest that the glioblastoma cells primarily use the mesenchymal motility mode for invasion and dissemination. Once there is formal confirmation that glioblastoma cells do not have the capacity to switch to an amoeboid movement, therapies designed to block integrin receptor-mediated cell migration are likely to have significant success in blocking dissemination of glioblastoma.

### 4.2. Novel Cytoskeletal Targets

The determining role of the actin cytoskeleton in the morphological and mechanical properties that are indispensable for mesenchymal cell migration suggests that the actin filament system represents a major potential target for antimesenchymal migration strategies. However, the ubiquitous expression and vital contribution of actin to all cells and tissues in the body mean that nonspecific disruption of the actin cytoskeleton would cause unacceptable and life-threatening side effects. A more useful approach would therefore be to target specific actin regulatory molecules that are either restricted in their tissue distribution or are functionally specialized. For example, the tropomyosin family of actin-associating proteins has emerged as critical regulators of cell migration [95, 198–201]. Association of individual tropomyosin isoforms with actin filaments is proposed to impart distinct properties of actin dynamics on the associated filament [202], and it has been suggested that this specialization of the actin filaments may represent rational targets for chemotherapy [203]. 

The dynamics of actin filaments are critical in cellular processes ranging from cell division to apoptosis and migration. The dynamics are therefore subject to regulation by a vast repertoire of actin-regulatory proteins. Actin-regulatory proteins that modulate actin function in cell migration include ADF/cofilin [204]. Cofilin is highly expressed in glioblastoma tumor cells and is positively correlated with motility [205, 206]. Furthermore, cofilin activity directly relates to invasiveness and metastasis in mammary tumors [207]. However, conflicting data [208] and the fact that cofilin inhibition is lethal to both normal and tumor cells [204] necessitate intervention with other components of the cofilin signaling pathway rather than complete cofilin shutdown. In this regard, proteins such as Phospholipase C-gamma (PLC*γ*), slingshot (SSH), LIMK, or chronophin may represent valid targets for anticancer treatment as each of these molecules is mediator of cofilin signaling. Approaches to inhibit or stabilize these pathway intermediates may suppress the pathway activity sufficiently to derive therapeutic benefit in the absence of unacceptably high toxicity levels in nontumor cells.

The Rho GTPases RhoA, Rac1, Cdc42, and Ras indisputably control signal transduction pathways that cooperate to promote cell movement [122]. Given the defining role of Rac GTPase activity in the mesenchymal mode of cell migration [209], it seems sensible that approaches targeting GTPase activity to block glioblastoma cell invasion and dissemination should focus on the inhibition of Rac1 activity as a priority. Rac1 activity controls *de novo* actin nucleation at the periphery of the cell, resulting in lamellipodia extensions and membrane ruffling [115, 210]. In human glioma cells the suppression of Rac1 activity induces apoptosis [211], a finding that raises the important concept that arresting cell motility pathways may influence a cancer cell's apoptotic response in a way which could be therapeutically exploited [212]. Indeed, in nontransformed cells there are many cellular safeguards to ensure that the apoptosis programme is initiated following detachment from the extracellular matrix [213]. These safeguards are subverted in oncogenically transformed cells [214, 215] allowing cells to survive the remodeling and turnover of adhesion sites that accompanies the progression to an invasive and migratory phenotype. Correlated to this, increased activation of the PI3K survival pathway in glioma cells [212] overcomes detachment-mediated apoptosis as these cells invade and migrate. An important caveat to the use of Rho GTPase inhibitors is the essential role they play in many different cellular functions and thus the potential for side effects in otherwise healthy tissues. Specific signaling functions of the Rho GTPases are directed in part by selective interactions with guanine nucleotide exchange factors that regulate GTPase activity. Such molecules may therefore represent attractive targets for drug intervention with fewer side effects [216].

The PI3K pathway contributes to cytoskeletal remodeling via signaling through the serine/threonine kinase Akt [217]. Importantly, migrating glioma cells show elevated levels of phosphorylated Akt and its substrate, Glycogen Synthase Kinase-3 (GSK-3) and phosphorylated Akt, localizes to the leading edge of migrating glioblastoma cells [212]. PI3K is activated by receptor tyrosine kinases through Ras, binding to the p110 subunit of PI3K [218, 219]. A major consequence of this is the generation of Phosphatidylinositol (3,4,5)-trisphosphate (PIP3) in the plasma membrane which in turn acts as second messenger to activate Akt and other proteins. PIP3 in the inner leaflet of the plasma membrane recruits and activates Akt [220, 221] which results in cell survival, proliferation (cell number), and/or growth (cell size) [217]. The conversion from PIP3 to Phosphatidylinositol (4,5)-bisphosphate (PIP2) is catalyzed by several phosphatases. Significantly this includes PTEN which is a tumor suppressor protein in glioblastoma and other cancers [20, 222, 223]. PTEN negatively regulates cell migration by downregulating Rac1 and Cdc42 via its lipid phosphatase activity by decreasing PIP3 levels [224, 225]. Furthermore, PTEN inhibits cell migration by downregulating FAK and p130Cas phosphorylation [24]. Pharmaceuticals that either downregulate PI3K/PIP2 or stabilize and/or upregulate the expression of PTEN might therefore decrease the rate of metastasis in treated cells. An experimental PI3K inhibitor, PX-866 (which is an improved wortmannin analogue), inhibits tumor cell migration at sub-nanomolar concentrations [226].

### 4.3. Approaches to Identifying Relevant Adhesion Signaling Proteins

Given the critical role of migration in the progression to metastatic, disseminated cancer, there are increasing numbers of studies taking a global approach to identifying molecules that are essential regulators of cell migration. In one example, investigators used an siRNA screening approach to identify proteins that modulate the migratory response of a breast epithelial cell line [227]. The investigators took advantage of the recently described adhesome [72] and restricted their siRNA targets to a set of “migration and adhesion related” gene targets. The advantage of such an approach over genetic profile analysis is that screening for functional outcome concurrently validates the target. Beyond such functional screens, the wealth of gene expression data now available [228–230] means that it is possible to identify potential adhesion and migration related proteins that are differentially expressed in glioblastoma and directly test these molecules. Alternatively, we can use our current knowledge of adhesion signaling pathways to predict likely candidate molecules. For example, based on the known interaction of FAK with the Cas family of proteins and their role in promoting cell migration it was proposed, and subsequently confirmed, that the Cas protein Neural precursor cell-expressed, developmentally downregulated 9 (NEDD9), also known as Human enhancer of filamentation 1 (HEF1) and Crk-associated substrate-lymphocyte type (Cas-L), is a specific regulator of glioblastoma cell invasion through brain homogenates [70], and it is now apparent that NEDD9/HEF1/Cas-L is a critical regulator of Rac-mediated mesenchymal cell migration [209]. The emerging role of NEDD9/HEF1/Cas-L as a key regulator of cancer metastasis [202] means that this molecule will increasingly be the target of novel anticancer treatments [231]. No matter which approach is taken to identify new cytoplasmic adhesion molecules that are essential regulators of glioblastoma invasion it is vital that each of these molecules is tested using models that faithfully recapitulate the brain environment, for example, in the form of orthotopic xenograft mouse models [231] or via the use of culture models such as the organotypic brain slice cultures [38, 39].

Finally, we note that the blood-brain barrier (BBB) constitutes a major hurdle for any systemic chemotherapy delivery to treat glioblastoma. The BBB protects the brain by excluding molecules by size and biochemical properties and generally prevents the translocation of lipophilic molecules larger than 500 Da to the brain [232–234]. However, Temozolomide, an antiglioblastoma drug which is already in clinical use, can successfully cross the BBB after oral administration [235, 236]. Thus any approaches to treat glioblastoma infiltration by targeting the molecular regulators of mesenchymal migration will need to address this issue. The field of drug targeting to the brain is currently actively under investigation in parallel with our increasing knowledge of the molecular regulators of mesenchymal migration. Avenues being pursued range from transient osmotic BBB disruption, nanoparticle carriers, to direct injection into the brain parenchyma [232, 234, 237–239]. Thus, we anticipate that combined efforts in these two research fields will facilitate the derivation of anti-infiltration drugs that are able to cross the BBB and successfully treat glioblastoma.

## 5. Conclusion

There is clearly an urgent need to develop new approaches to treating glioblastoma—the current poor rate of survival means that even small improvements in therapy have the potential to significantly improve life expectancy and/or quality of life for patients diagnosed with this tumor. In the present paper, we have highlighted recent understanding of the cellular mechanisms that regulate mesenchymal cell migration that is characteristic of infiltrating glioblastoma. New targeted therapies focusing on the unique cell biology that facilitates infiltration of the brain parenchyma offer promise to treating patients with glioblastoma. Understanding cell migration is a necessary first step in developing anti-migration pharmaceuticals. It is unlikely that any agent targeted against infiltration will be effective unless used in combination with therapies that target other aspects of tumor biology. Current clinical trials generally measure improvements in time to progression, but overall impact on long-term survival has been modest. The challenge for the future is to translate migration and invasion research into new therapies for use in the clinical setting and to the design of clinical trials including outcomes and markers that report successful inhibition of infiltration. As our understanding of the cell machinery that regulates migration continues to improve apace, the possibility of improved patient outcomes by pursuing novel targeted therapies moves forward towards reality.
